# Tool-mediated patienthood: structural plurality in oncology, surgery, and palliative care

**DOI:** 10.1007/s11019-026-10359-x

**Published:** 2026-05-11

**Authors:** Karen Yan, Min-Jung Cheng, Yun-Ying Kuo, Yong Alison Wang

**Affiliations:** 1 Institute of Philosophy of Mind and Cognition, National Yang Ming Chiao Tung University, No. 155, Section 2, Linong St, Beitou District, Taipei City, 112 Taiwan; 2 Department of Internal Medicine, National Taiwan University Hospital, No. 7, Section 1, Zhongshan S. Rd., Zhongzheng Dist., Taipei City, 100 Taiwan; 3 Department of Philosophy, University of South Carolina, Close-Hipp, 1705 College St, Suite 503, Columbia, SC 29201 USA; 4 Department of Internal Medicine, Koo Foundation Sun-Yat Sen Cancer Center, 125 Lih Der Rd, Beitou District, Taipei City, 11259 Taiwan; 5 School of Medicine, National Yang Ming Chiao Tung University, No. 155, Section 2, Linong St, Beitou District, Taipei City, 112 Taiwan

**Keywords:** Patient-centered care, Autonomy, Shared decision-making, Philosophy of medicine, Cognitive tools, Ethnography of medicine, Structural plurality, Tool-first approach

## Abstract

Patient-centered care (PCC) is widely endorsed in contemporary medicine, yet philosophical analyses often approach it through concept-first approaches that define patienthood in advance—typically in terms of autonomy, holistic personhood, or rational agency—and then assess clinical practice by reference to these ideals. This paper argues that such an approach can obscure how patienthood is configured in practice. We develop a tool-first approach that treats cognitive, communicative, and material tools as analytically primary for understanding how patients are individuated in clinical reasoning. The argument is grounded in an ethnographic case study conducted in a specialized cancer hospital, focusing on outpatient clinics in medical oncology, colorectal surgery, and palliative care. Rather than treating ethnography as descriptive background, we use it to identify tools-in-use that structure what becomes salient, actionable, and patient-relevant in situated encounters. Across these settings, distinct configurations of tools generate systematically different modes of patienthood. In oncology, staging systems and expectation management configure patients as therapeutic trajectories oriented toward uncertain futures. In surgery, anatomical diagrams and probabilistic framings individuate patients as operative bodies embedded in structured decision spaces. In palliative care, symptom scales, narrative practices, and informational scaffolding configure patients as experiential subjects and epistemic agents. We analyze these differences as instances of structural plurality: patterned, tool-mediated modes of patient individuation that are internally coherent yet irreducible to a single model. On this account, ethical ideals commonly associated with PCC—such as autonomy, shared decision-making, and informed consent—can be understood less as prior normative standards applied to practice, and more as contingent achievements that depend on how tools structure salience, understanding, and possibilities for agency in clinical contexts.

## Introduction

Patient-centered care (PCC) is widely endorsed as a core value in contemporary medicine and is frequently described as an effort to treat patients as individuals rather than as mere cases of disease (Institute of Medicine 2001; Mead and Bower 2000; Epstein and Street 2011). Central to this commitment is an implicit concern with patienthood: what it means to be a patient, what features of patients ought to matter for clinical reasoning, and how care should be organized in response. Across clinical ethics, health policy, and medical practice, PCC functions as a normative orientation that links conceptions of patienthood to judgments about what counts as appropriate, respectful, or ethically adequate care.

In the broader clinical and bioethics literature, PCC is articulated through several overlapping frameworks. These include the biopsychosocial orientation, which emphasizes the integration of biological, psychological, and social factors (Engel 1977; Mead and Bower 2000); the patient-as-person tradition, which highlights the moral significance of attending to patients’ lived experience (Cassel 1982; Mead and Bower 2000; Carel 2016); the therapeutic alliance model, which stresses relational engagement between clinician and patient (Epstein and Street 2011); and normative commitments to autonomy, shared decision-making, and informed consent that structure much contemporary clinical ethics (Beauchamp & Childress 2019; Stiggelbout et al. 2012). Although these approaches differ in emphasis, they share a common orientation: patienthood is typically defined in advance through normative or conceptual ideals. Patients are described as rational decision-makers, holistic psychosocial beings, or rights-bearing agents, and clinical practices are then evaluated by reference to how closely they approximate these prior characterizations of patienthood (Mead and Bower 2000; Scholl et al. 2014; Stiggelbout et al. 2012). We refer to this orientation as a concept-first approach to patient-centered care.

Our claim is not that these literatures are identical in their ethical commitments or practical aims. They differ substantially in whether they foreground psychosocial integration, relational engagement, or decisional rights. What unites many of them for present purposes is a narrower methodological tendency: they specify patient-relevant features in advance and then assess practice in relation to those prior specifications.

Within this framing, variation across clinical settings is commonly interpreted as deviation from, or incomplete realization of, a general normative model of patienthood. Yet when PCC is examined in clinical practice, what counts as centering the patient does not consistently take the form of more or less successful instantiations of a single ideal. Across different contexts, patient-centered care may emphasize biomedical individualization, standardized procedures, or deliberative dialogue (Epstein et al. 2005; Scholl et al. 2014). Rather than converging on a stable model of patienthood, these patterns suggest a more systematic form of variation. One way to read this variation is not as evidence of conceptual instability or normative failure, but as raising a question about how patienthood itself is being engaged across settings: whether different clinical contexts may be organizing and responding to patients in meaningfully different ways.

To address this question, we draw on resources from the philosophy of scientific practice. Over the past two decades, philosophers of science have increasingly adopted a practice-first orientation, treating scientific practice rather than abstract theory as the primary unit of analysis (Mitchell 2003; Kellert, Longino, and Waters 2006; Chang 2004; Ankeny & Leonelli 2016, 2020; Guay & Pradeu 2015; Fagan 2015; Chen 2015; Yan & Hricko 2017; Bueno, Chen, and Fagan 2018; Chen & Hricko 2019; Nersessian 2022; Chang 2022; Massimi 2022). This body of work has shown how scientific objects, norms, and forms of reasoning are shaped through material, cognitive, and social practices. At the same time, it raises a methodological question central to practice-based inquiry: once practice becomes the focus of analysis, how are its components to be individuated in a way that supports explanation and comparison, rather than remaining at the level of descriptive richness alone? In response to this methodological question, we propose what we call a tool-first approach. Rather than beginning with a fixed conception of patienthood, this approach examines how cognitive, normative, and material tools structure clinical reasoning in situ. On this view, patienthood is not simply recognized by reference to a prior concept, but is organized through the tools clinicians employ. Our claim is not that tools bring patients into existence simpliciter. It is that tools are constitutive in a more limited analytic sense: they help determine what counts as the patient for clinical reasoning, action, and evaluation in a given setting. Patient-centered care thus provides a particularly revealing case for examining how different forms of patienthood are made salient and actionable in practice.

To examine how a tool-first approach can be developed in practice, we conducted an ethnographic case study of cancer care in a specialized hospital, focusing on outpatient clinics in oncology, surgery, and palliative care. Across 125 h of field observation and 17 in-depth interviews with clinicians and patients, we examined how tools-in-use structure clinical reasoning and shape what counts as the patient. Our analysis identifies three recurrent modes of patient individuation: therapeutic trajectories in oncology, operative bodies in surgery, and experiential subjects and epistemic agents in palliative care. These models should be read as analytic reconstructions of recurrent tendencies within a bounded case study. They do not describe every consultation, exhaust each specialty, or preclude overlap and variation within or across settings.

We suggest that approaching PCC through a tool-first lens offers a way of understanding its plurality without reducing it either to conceptual ambiguity or to normative failure. Instead of evaluating practice solely by reference to pre-given ideals of patienthood, this approach invites attention to how specific tools organize salience, agency, and understanding in clinical encounters.

The paper proceeds as follows. Section "The concept-first approach to patient-centered care: individuating through pre-existing concepts" examines how existing accounts of PCC exemplify the concept-first orientation and outlines its limitations. Section "A qualitative case study in a cancer care hospital" presents our ethnographic methodology. Section "The tool-first approach to PCC: individuating patienthood via tools-in-use" analyzes our empirical material to show how different tools organize and stabilize different forms of patienthood in practice. Section "Tool-first rethinking of patient-centered care" explores the normative implications of this analysis for patient-centered care. Section "Conclusion" concludes by drawing broader metaphilosophical lessons for the analysis of scientific and clinical practice.

## The concept-first approach to patient-centered care: individuating through pre-existing concepts

PCC is widely endorsed as a normative commitment in contemporary medicine and is promoted in policy, clinical guidelines, and patient advocacy (Institute of Medicine 2001; Epstein and Street 2011). At a general level, it is often characterized as an effort to recognize patients as individuals rather than as mere cases of disease (Mead and Bower 2000). Yet despite this broad consensus, PCC has proven difficult to specify in a stable and unified way. In both philosophical and clinical discussions, attempts to give PCC determinate content frequently proceed by adopting a concept-first approach: beginning with a fixed conception of patienthood or personhood and then evaluating clinical practice according to how closely it conforms to that conception.

The limitations of this approach can be illustrated through three recurrent challenges.

First, ambiguity. PCC is widely invoked as a guiding ideal, yet its meanings vary across contexts. In some settings, it refers to biomedical individualization, such as tailoring treatment based on genetic or physiological differences; in others, it denotes shared decision-making and respect for patient autonomy; elsewhere, it foregrounds social determinants of health (Epstein et al. 2005; Scholl et al. 2014). In many concept-first discussions, this diversity of meanings is treated as a problem of conceptual clarity to be resolved through more precise definitions. What this response leaves underexamined is the possibility that such diversity reflects differences in how patienthood is being engaged and organized in practice, rather than instability at the level of concepts.

Second, standardization versus individualization. Policymakers and institutions often seek to institutionalize PCC through checklists, performance metrics, and reimbursement schemes (Berwick 2009). Yet when clinical work is organized primarily around standardized protocols, patient-centeredness risks becoming procedural rather than substantively responsive (Greenhalgh et al. 2014). From a concept-first perspective, this tension arises because efforts to operationalize PCC rely on fixed criteria intended to apply across settings.

Third, the role of expertise. PCC emphasizes autonomy and shared decision-making, yet in practice, conflicts between patient preferences and clinical judgment are common (Stiggelbout et al. 2012). Patients may request interventions that clinicians judge to be of limited benefit, or may lack the information needed to articulate stable preferences. In such situations, pre-existing conceptions of the autonomous or holistic patient offer limited guidance for navigating how reasoning should proceed in situ. What is at issue is not the irrelevance of these concepts, but their underdetermination with respect to the situated demands of clinical decision-making.

Taken together, these challenges point to a philosophical limitation of concept-first approaches. When patients are individuated primarily through pre-given notions of patienthood or personhood—such as rational decision-maker, embodied organism, or psychosocial whole—plurality across clinical settings is likely to be interpreted as deviation from an assumed unity. What is obscured, on this reading, is the possibility that plurality may be a constitutive feature of practice rather than a failure of conceptual coherence. The contrasts invoked here—such as those between patients understood as individuals and patients treated as cases of disease, or between unity and plurality—are intended as analytic devices for tracing how different conceptual framings specify what counts as patient-relevant, rather than as exhaustive definitions or ontological dichotomies.

This difficulty is not confined to medicine. In several areas of philosophy of science, influential critiques of traditional epistemology have drawn attention to the limits of concept-first strategies when confronted with plurality in scientific practice. For example, Longino’s analyses of contextual values in science argue that objectivity cannot be secured by appeal to a single abstract ideal, but must instead be understood through the ways epistemic norms are sustained within particular social and institutional settings (Longino 1990, 2002). Nersessian’s studies of model-based reasoning likewise emphasize that scientific cognition cannot be adequately characterized by one general account of representation, since different modeling practices support distinct forms of reasoning (Nersessian 2008). Chang’s work on epistemic iteration further highlights how scientific progress depends on historically situated tools and categories rather than on convergence toward a single conceptual essence (Chang 2004).

These discussions suggest that plurality in scientific practice is not a defect to be eliminated at the conceptual level, but an outcome of how inquiry is organized through heterogeneous tools, norms, and practices—an insight that has motivated practice-first approaches in philosophy of science.

At the same time, practice-first analysis raises its own methodological question. While close attention to practice can illuminate situated reasoning, it does not by itself specify which elements of practice are analytically salient or how plurality should be systematically individuated (Chen 2015; Chang 2004, 2012, 2022; Bueno, Chen, and Fagan 2018; Ankeny & Leonelli 2020; Nersessian 2022; Massimi 2022). In the absence of such a strategy, ethnographic richness risks remaining descriptively informative without offering clear criteria for philosophical comparison or generalization.

The tool-first approach proposed here is intended as one way of addressing this methodological gap. Rather than presupposing a concept of patienthood or remaining at the level of description, it examines how tools-in-use structure reasoning and interaction by foregrounding some features and backgrounding others. By attending to cognitive, normative, and material tools, philosophers can analyze how different forms of patienthood are configured in practice without reducing plurality to conceptual deviation.

This methodological reframing also helps clarify why PCC, when translated into practice, is frequently associated with recurring ethical tensions. While concept-first ideals have shaped influential models of PCC, their enactment in clinical settings has been widely noted to generate tensions in practice. Autonomy can be complicated by prognostic uncertainty, where withholding or reframing information may sustain therapeutic engagement while simultaneously limiting informed choice. Shared decision-making, often promoted as a gold standard, is constrained by informational asymmetries and by the structuring role of clinical tools that prefigure which options can meaningfully be compared. Informed consent, formally central to biomedical ethics, rarely operates as a discrete event; in areas such as oncology and palliative care it often unfolds as a continuous negotiation, mediated by metaphors, narratives, and symptom scales.

These observations motivate a methodological demand that guides the remainder of the paper: how can philosophers identify the tools that structure clinical practice without presupposing a fixed concept of patienthood?

## A qualitative case study in a cancer care hospital

If the concept-first approach falters because it presupposes unity at the level of patienthood, then a tool-first analysis requires a different strategy: it must begin from how tools are actually used in practice. This methodological demand has epistemic as well as philosophical consequences.

Tools-in-use cannot be reliably identified through armchair theorizing or by consulting clinical guidelines alone, since many such tools are tacit, embodied in interactional routines, or embedded in unarticulated patterns of reasoning. To individuate practice through tools, therefore, it is necessary to observe practice in situ and attend to the cognitive scaffolding that clinicians themselves deploy.

This is why our project combines philosophy of science with ethnographic inquiry. Ethnography, in this context, is not introduced as a sociological supplement but as a method especially well-suited to identifying tools-in-use without presupposing a fixed conceptual model of patienthood.

We do not claim that ethnography is methodologically necessary for every inquiry into patient-centered care. The stronger claim is narrower: for our specific question—how tacit tools-in-use scaffold clinical reasoning in live encounters—guidelines, policy documents, and retrospective interviews are insufficient on their own, because they more readily capture avowed norms than enacted cognitive organization. If philosophy of science aims to understand practice from within, then philosophers must attend to how reasoning unfolds in real time. Ethnographic methods provide access to tacit strategies, communicative habits, and informal reasoning patterns that constitute the cognitive architecture of practice but rarely surface in formal guidelines or retrospective accounts.

At a more epistemological level, ethnographic observation plays a necessary evidential role here not because philosophical analysis requires empirical validation, but because the object of analysis is reasoning as it unfolds in practice. The tool-first approach is concerned with how clinical reasoning is scaffolded by publicly enacted cognitive, normative, and material resources—such as staging systems, diagrams, scales, and communicative routines—rather than with clinicians’ private mental states. These resources are accessible precisely through observation of interaction and practice, where their role in structuring salience and guiding action becomes visible. Ethnographic detail thus functions as philosophical evidence insofar as it reveals recurrent, stabilized patterns in how tools organize reasoning and individuate patients in situ.

Yet our use of ethnography differs in purpose from its role in science and technology studies (STS). In STS, ethnography often aims to illuminate the social, institutional, and cultural conditions of scientific work. Our project, by contrast, employs ethnography instrumentally to address a philosophical question: how tools structure clinical reasoning and individuate patients in practice. Interviews and observations are not treated as data for sociological claims, but as occasions to identify the cognitive, normative, and material resources through which patienthood is configured in practice.

This orientation requires methodological reflexivity. Philosophers engaged in ethnography must translate across levels: from the micro-details of clinical encounters, to the meso-level of recurrent tools and practices, and finally to the meta-level of philosophical analysis. Without ethnographic immersion, policy documents or ethical ideals might too readily be taken as the primary drivers of clinical reasoning. Our observations instead indicate that reasoning in situ is structured by staging systems, risk estimates, symptom scales, and communicative strategies—resources that would likely remain invisible without close observation.

With this orientation in place, we conducted a qualitative case study in a specialized cancer hospital that explicitly endorses patient-centered care. We selected this site because its institutional mission foregrounds PCC, making it a suitable setting for examining how patient-centeredness is realized in practice. Within this single institutional context, we focused on three outpatient clinics—medical oncology, colorectal surgery, and palliative care—to explore how patienthood is individuated across distinct domains of cancer care.

To operationalize this metaphilosophical agenda, we employed a three-step methodological design:

Step 1: Situated observation of clinical encounters. Over 125 h of team-based ethnographic fieldwork, three researchers observed the same consultations simultaneously, recording independent field notes that were later compared for convergence. This step allowed us to explore recurring patterns of salience—what clinicians consistently foregrounded or backgrounded when engaging patients.

Step 2: Elicitation of reasoning through interviews. Based on our field observations, we designed interview questions for clinicians and patients that probed how decisions were justified, what counted as relevant, and how participants made sense of their encounters. In total, we conducted 17 interviews (9 clinical professionals, 8 patients). This step enabled us to uncover the tacit cognitive tools guiding action, which often remained implicit during live interactions.

Step 3: Iterative philosophical analysis. We did not treat the data as self-explanatory, nor as raw material for sociological description. Instead, we analyzed it through the lens of the tool-first approach: asking what cognitive resources clinicians mobilize, how these resources structure reasoning, and how they foreground or background different features of patient encounters. This analytic step transformed ethnographic detail into philosophical evidence, enabling us to identify implicit reasoning structures without presupposing a fixed concept of patienthood.

Taken together, this design illustrates how empirical methods can serve a metaphilosophical purpose. Rather than importing ready-made concepts of patienthood, we use ethnography to identify the tools through which patients are individuated in practice. Ethnographic observation is especially important for detecting tools-in-use as they operate in real time, thereby providing the evidential basis for philosophical analysis of individuation that armchair reflection alone cannot supply.

This methodological choice is not merely ancillary to philosophical analysis but central to the present argument. For philosophers of medicine, attending to tools-in-use makes visible how autonomy, consent, and shared decision-making function not as abstract ideals but as fragile accomplishments sustained—or undermined—by the architectures of practice. In the next section, we apply this methodological orientation to our empirical material, showing how distinct tools organize different forms of patienthood in oncology, surgery, and palliative care.

## The tool-first approach to PCC: individuating patienthood via tools-in-use

The preceding critique of the concept-first approach suggested that pre-given notions of patienthood are limited in their ability to account for the plurality of clinical reasoning. A tool-first approach, by contrast, begins with the resources clinicians actually employ and examines how these resources structure what counts as the patient in practice. This section shows how such an analysis can be developed on the basis of empirical material.

To illustrate this approach, we draw on our single-center ethnographic case study of oncology, surgery, and palliative care clinics. In this setting, we recurrently observed that clinicians relied on implicit cognitive tools to guide how they engaged with patients. These tools were rarely made explicit in formal training or documentation; instead, they became visible through patterns of reasoning, interaction, and communication. By ‘patient model’ we mean the patterned way in which a particular clinical setting makes a certain kind of patient salient for reasoning and action. Understood in this sense, patient models function as tools-in-use: they foreground some features of the clinical encounter, background others, and thereby shape how patient-centeredness is enacted.

In our case study, we identified three recurrent patient models:

1. In medical oncology, patients were framed as therapeutic trajectories shaped by staging systems and expectation management.
2. In colorectal surgery, patients appeared as operative bodies with calculable risks, structured by anatomical diagrams and quantified comparisons of procedures.
3. In palliative care, patients were individuated as experiential subjects and epistemic agents, through symptom scales, narrative recapping, and informational exchange.

It is worth noting that the models discussed below are not intended as exhaustive descriptions of entire specialties, but as analytic characterizations of recurrent tool-mediated patterns observed within this institutional case. In what follows, we present each model in turn. Section "The patient model of the medical oncologist" describes how these patient models are enacted through tools-in-use in clinical encounters, while Sect. "The patient model of the colorectal surgeon" develops their philosophical significance by analyzing how different modes of individuation are generated through these tools.

### The patient model of the medical oncologist

**Fig. 1:**
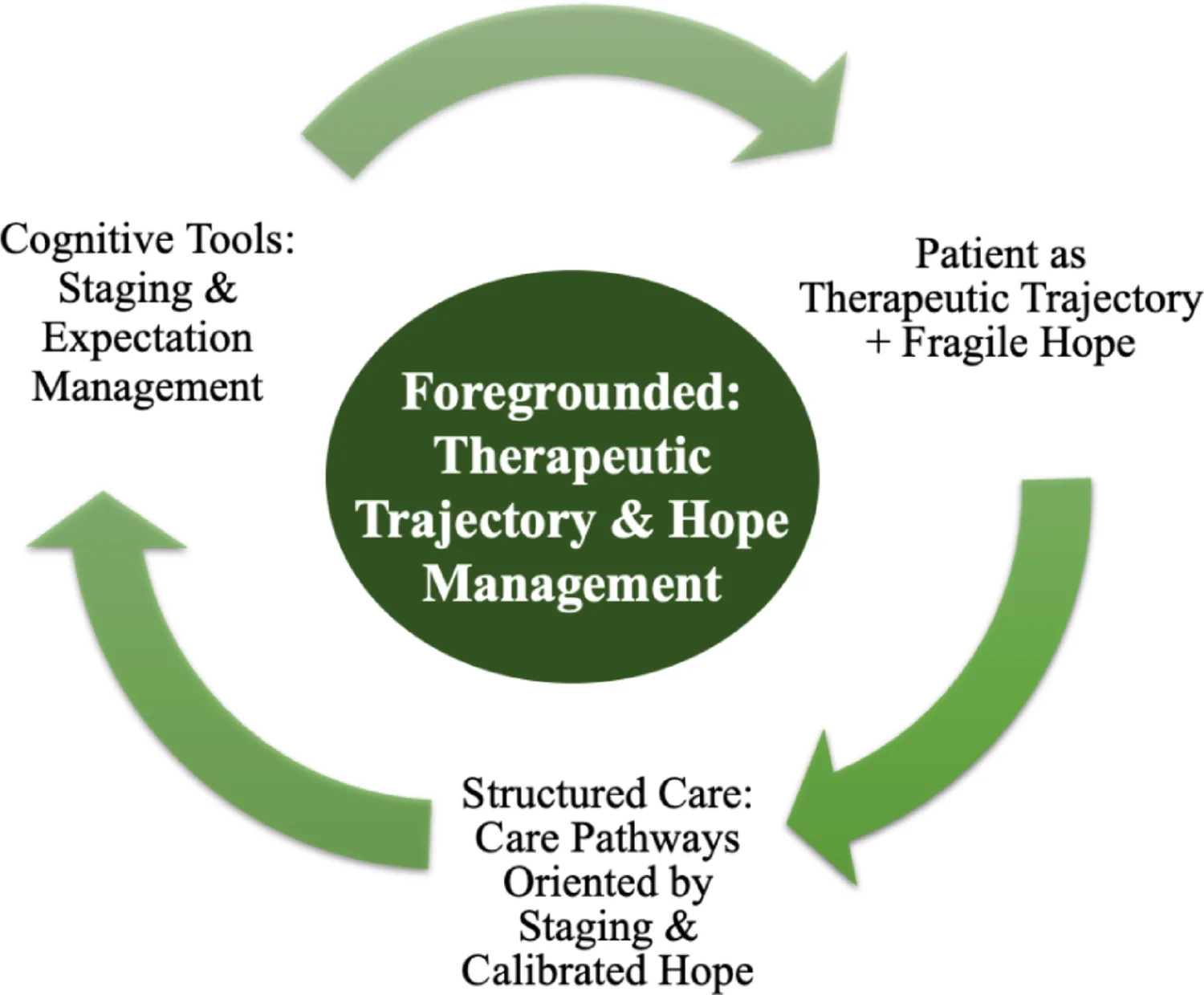
Patients are individuated as therapeutic trajectories and bearers of fragile hope. Staging and expectation management foreground therapeutic trajectory and hope, structuring care as pathways oriented by staging and calibrated hope. The cyclical arrows indicate that these processes are not linear but iteratively recalibrated in practice

Figure 1 illustrates the patient model implicit in medical oncology practice. Here, patients are individuated as therapeutic trajectories, structured by staging systems and communicative strategies of expectation management.

The staging system was the primary tool through which oncologists individuated patients. Each consultation began by locating the patient within a formalized trajectory of disease progression: newly diagnosed or recurrent, stage I through IV. This tool established not just a diagnostic category but a temporal horizon. Our field notes document that consultations often opened with references such as stage III or stage IV recurrent, which immediately situated the patient within a broader clinical pathway of treatment options and anticipated prognosis. In this way, staging did not simply classify but actively temporalized patienthood—rendering the patient as a point along a projected course of illness.

Expectation management complemented this temporal individuation. Rather than offering precise survival probabilities, oncologists framed prognoses in cautiously optimistic terms. This practice, as one oncologist explained, reflected the difficulty of sustaining therapeutic engagement:

They [the patients] hope to get rid of the disease…even though some of them realized that it’s impossible, they still express that they want nothing less than being cured. If that is not attainable, the patients will have complaints of disappointment and we [the doctors] feel that we fail in our duty (M3, interview, 2022/07/29).

Here, patienthood is shaped not only by biomedical staging but also by an affective economy of hope. Our ethnographic observations show that oncologists continually navigated between the formal temporality of staging and the fragile temporality of expectation (a precarious future-orientation that sustains therapeutic hope but is easily unsettled by new information). Patients were thus individuated simultaneously as trajectories of disease and as bearers of hope—two dimensions that could reinforce or strain against one another.

As Fig. 1 depicts, the oncologist’s patient model individuates patients as therapeutic trajectories, organized by staging systems and modulated by the management of hope. Patient-centeredness here means aligning care with the temporal logic of disease progression while sustaining the fragile affective conditions under which patients remain engaged with treatment.

By refusing to quantify prognosis, the oncologist sustains therapeutic engagement under conditions of uncertainty. Yet this practice also reconfigures the ethics of disclosure: what appears as beneficent withholding from one perspective may equally be seen as a constraint on autonomy. The very tools that make hope sustainable thus also generate tensions about how autonomy can be respected in the face of radical uncertainty.

Surgical consultations, by contrast, framed patienthood in an entirely different register.

### The patient model of the colorectal surgeon

**Fig. 2:**
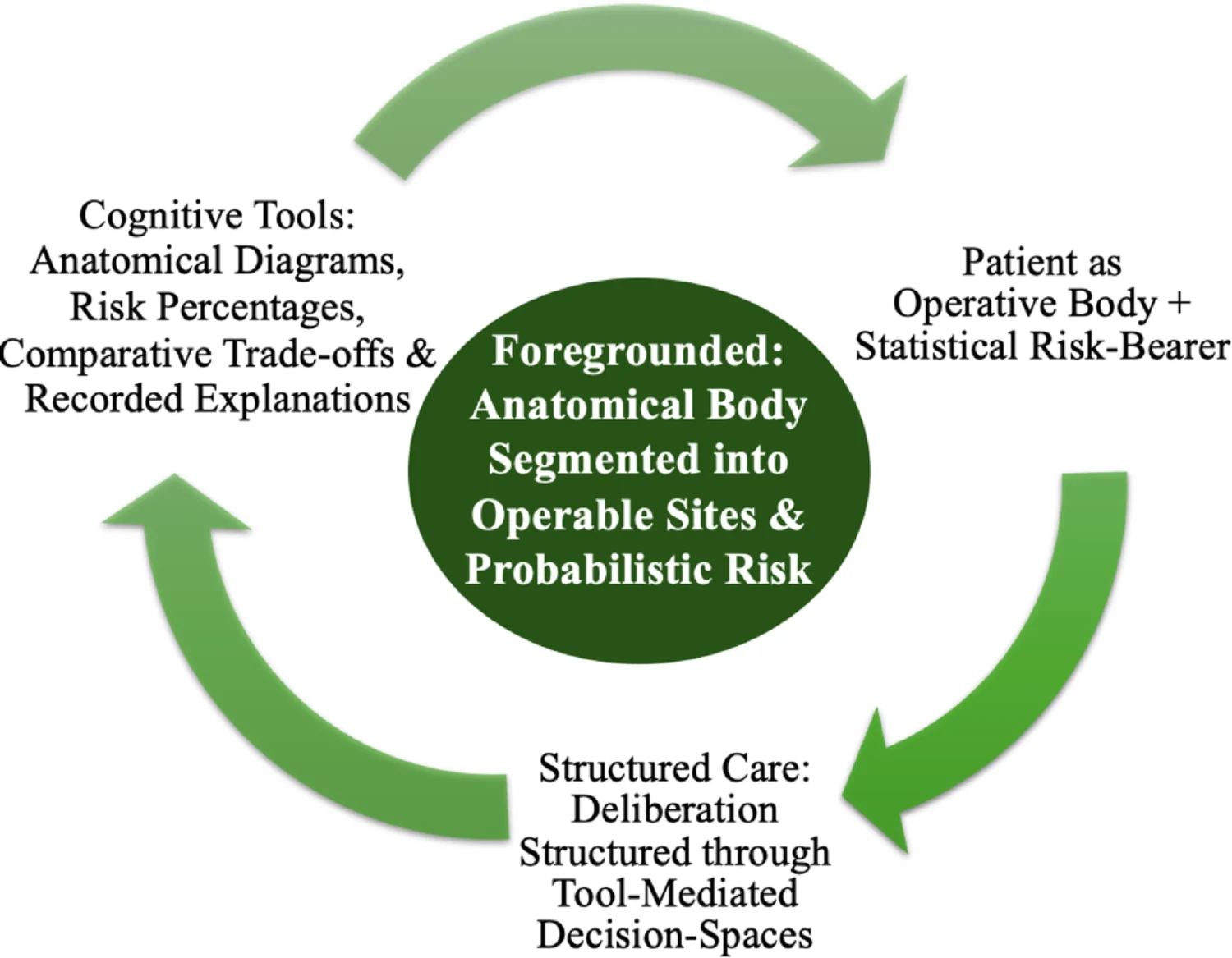
Patients are individuated as operative bodies and statistical risk-bearers. Anatomical diagrams, quantified risks, and comparative explanations foreground the body as segmented and probabilistic, structuring care as decision-spaces that enable deliberation. The cyclical arrows indicate that these processes are not linear but iteratively recalibrated in practice

Figure 2 illustrates the patient model implicit in colorectal surgery practice. Here, patients are individuated as operative bodies with calculable risks, structured by anatomical diagrams, quantified probabilities, and systematic comparisons of procedures.

Anatomical visualization was the first and most consistent tool. When patients came to consult the surgeon about operative options, the encounter typically began with the surgeon sketching simplified diagrams of the colon, rectum, and adjacent lymph nodes. These drawings were used not only to explain the patient’s condition but also to show how the surgery would be performed, thereby framing the patient as a surgical body composed of localized anatomical parts. This practice contrasted sharply with the oncologist’s reliance on staging: while oncology situated patients on a disease trajectory, surgery individuated them as sites of operative intervention.

Risk quantification provided the second structuring tool. Unlike oncologists, who avoided providing precise survival figures, surgeons openly framed decisions in probabilistic terms. As recorded in our field notes, the surgeon explained to a patient:

The risk of having leakage after colon anastomosis is around 5–10%.

The rate of converting from laparoscopic surgery to open surgery is 10%. (Field note, 2022/04/20)

Such probabilistic framing did not function merely as background information but as a scaffold for deliberation. Our ethnographic observations showed that patients and families oriented their questions around these numbers, using them as anchors for imagining possible outcomes and weighing alternatives. In this sense, risk figures reconfigured agency: patients were not invited to deliberate in the abstract, but to situate their choices within thresholds of probability defined by surgical reasoning.

Finally, the surgeon systematically scaffolded deliberation by presenting comparative trade-offs between procedures. In discussions of robot-assisted surgery, for example, the advantages of precision, smaller incisions, and faster recovery were weighed against higher cost and technical limitations. Beyond this, the surgeon also encouraged patients and families to record consultations:

It’s self-contradictory to hope the patient and their family fully understand the surgery while limiting their repeat access to the consultation. Recording helps them make informed decisions together. (M1, interview, 2022/07/07)

This unusual practice highlights that patient agency in surgery was not grounded in abstract appeals to autonomy, but in communicative tools that structured the decision-making process.

As Fig. 2 depicts, the surgeon’s patient model individuates patients as operative bodies parsed through anatomy and risk, whose agency emerges within a structured field of probabilities and comparative options. Patient-centeredness here consists not in applying a fixed concept of autonomy, but in enabling deliberation through the very cognitive tools that surgery makes available.

The probabilistic rendering of risk and the comparative structuring of options scaffold the very possibility of deliberation. But these same tools also delimit what can count as a patient decision, reducing agency to the navigation of predefined statistical trade-offs. Shared decision-making here is not neutral facilitation but a practice structured through numerical framings of risk, which both constrain and enable autonomy.

Palliative care, in turn, foregrounded yet another mode of individuation.

### The patient model of the palliative care physician

**Fig. 3:**
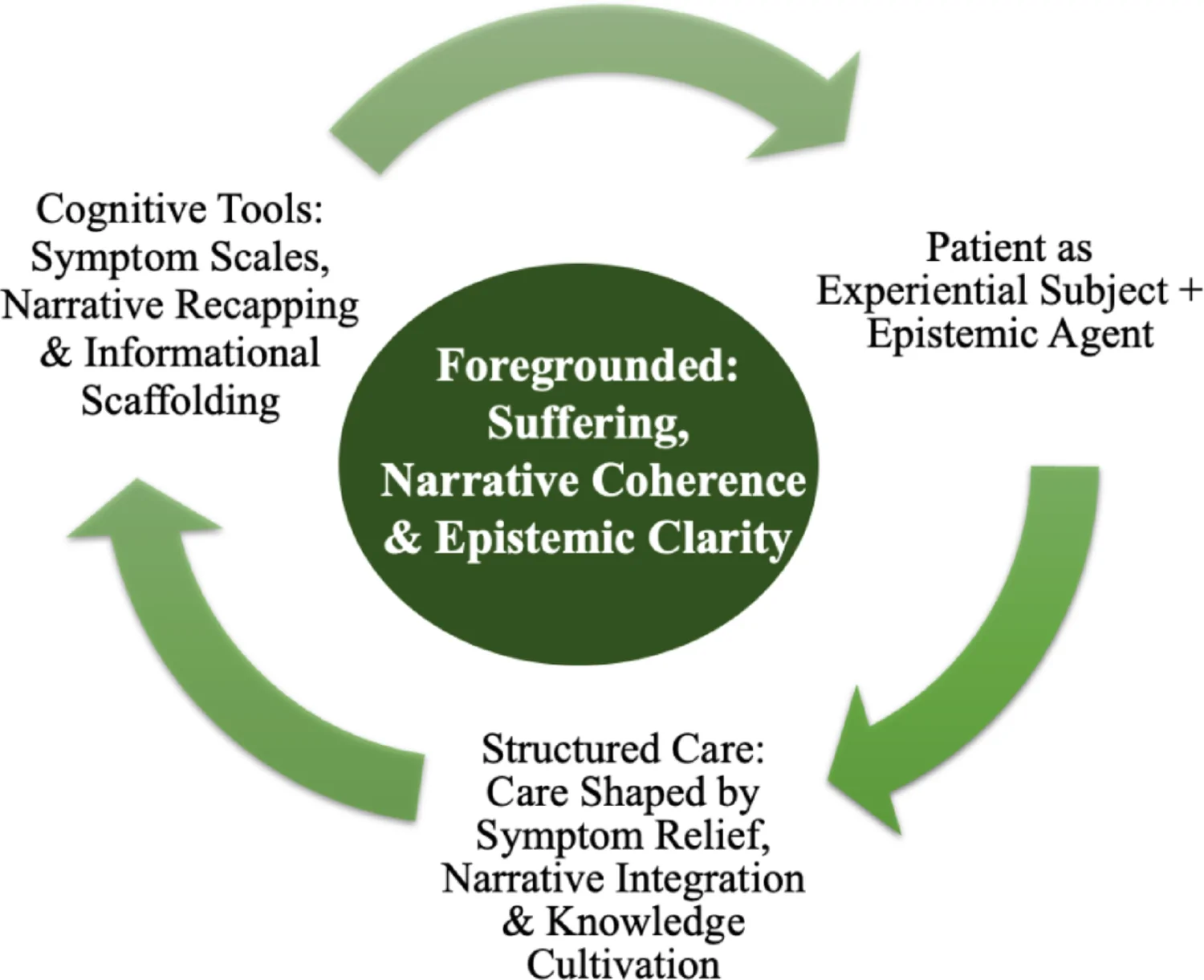
Patients are individuated as experiential subjects and epistemic agents. Symptom scales, narrative recapping, and informational scaffolding foreground suffering, coherence, and epistemic clarity, structuring care through symptom relief, narrative integration, and knowledge cultivation. The cyclical arrows indicate that these processes are not linear but iteratively recalibrated in practice

Figure 3 illustrates the patient model implicit in palliative care practice. Here, patients are individuated as experiential subjects and epistemic agents, structured by symptom-assessment tools, narrative practices, and informational scaffolding.

The first and most immediate structuring tool was symptom management. Unlike oncologists or surgeons, who centered care on disease control or operative success, the palliative care consultation foregrounded the alleviation of pain, fatigue, insomnia, and other forms of suffering. To support this, the clinician employed the Edmonton Symptom Assessment Scale (ESAS), a 1–10 rating system that enabled patients to express the severity of their symptoms numerically. As one practitioner explained:

One of the foremost goals of palliative care is to increase patients’ physical wellbeing by better controlling the symptoms they’re suffering from. (M2, interview, 2022/06/24)

In practice, ESAS was most clearly used during initial visits as a structured intake tool. In subsequent consultations, symptom management proceeded primarily through targeted questioning and pharmacological adjustments. While it is possible that clinicians also drew on ESAS scores during their private case evaluations, our interviews did not explicitly probe this longitudinal usage, and thus we refrain from stronger claims. What is clear, however, is that ESAS functioned to individuate patients at the outset as bearers of modifiable symptoms, establishing a clinical focus on what could be tracked, alleviated, and recalibrated.

A second structuring tool was narrative recapping, through which the physician systematically revisited each patient’s diagnostic and treatment history. This practice, often used to open the first consultation, helped patients reconstruct their illness trajectory and facilitated trust. In his words:

Journey-recapping is a useful strategy that could not only break the ice with the patient but also gain the patient’s trust by letting them know that the physician is well-prepared and understands their medical history. (M2, interview, 2022/06/24)

By reframing illness as a coherent story, this practice individuated the patient not only as a biological body but as a narrative subject, whose experience required interpretive integration as much as clinical management.

The third structuring tool involved informational scaffolding. Many patients arrived in palliative care without full knowledge of their medical status, sometimes still hoping for curative surgery despite widespread metastasis. The physician considered this epistemic gap itself a source of suffering:

Even in this hospital, many of my patients are still not informed about their medical condition when they come to me…The uncertainty of medical information definitely decreases the well-being of these patients. (M2, interview, 2022/06/24)

To address this, the practitioner engaged in detailed explanations of imaging scans, clarified treatment rationales, and prepared patients for anticipated decline. These practices positioned patients as epistemic agents whose understanding—of past decisions, present symptoms, and future prospects—was central to their care.

As Fig. 3 depicts, the palliative physician’s patient model individuates patients as experiential subjects shaped by symptoms, narratives, and knowledge. Patient-centeredness here means not recognizing a pre-given concept of autonomy, but constructing care around the management of suffering, the coherence of illness stories, and the cultivation of epistemic agency.

Narrative recapping and informational scaffolding position patients as epistemic agents capable of integrating experience and preparing for decline. Yet these same tools can risk paternalism, as the drive to correct misconceptions or provide anticipatory guidance may override patients’ own interpretive frames. Here the challenge is not only to manage suffering but also to enact informed consent as an ongoing process of communication, rather than a single event.

### Individuating patients via tools-in-use

The preceding analysis of oncology, surgery, and palliative care offers more than a set of ethnographic contrasts. It suggests that patients are not simply pre-existing entities awaiting recognition; rather, cognitive tools help organize what counts as the patient in practice. What is at issue is not only that clinicians emphasize different aspects of patient life, but that the tools they employ generate different modes of patient individuation. From the perspective of the philosophy of science, this implies that patienthood is not best understood as a single, universal object across settings, but rather as a family of situated constructions. To analyze this multiplicity, we therefore examine how tools perform the work of individuation: filtering salience, structuring reasoning, and shaping what counts as patient-centered care. Whereas the preceding sections focused on the empirical enactment of patient models within each setting, the analysis that follows abstracts from those descriptions to compare how different configurations of tools structure individuation and agency.

#### Medical oncology: trajectory-oriented individuation and tool-mediated agency

In medical oncology, patient individuation is organized around what can be analytically characterized as a trajectory-oriented model. The staging system functions as a primary filter of salience, organizing clinical attention along a temporal arc of disease progression. Within this framework, symptoms, concerns, and uncertainties are rendered patient-relevant insofar as they can be situated within the logic of staging and treatment sequencing.

Expectation management operates alongside staging to modulate the form of agency that becomes possible for patients. Rather than treating prognostic information as something to be transparently disclosed, oncological reasoning frequently involves calibrating hope in ways that sustain therapeutic engagement under uncertainty. From an analytic perspective, this practice configures patients not merely as biological carriers of disease, but as temporal agents whose agency is shaped through the management of uncertainty over time.

Philosophically, this mode of individuation complicates concept-first ideals of autonomy. Normative accounts often presuppose that autonomous choice depends on transparent information and stable preferences. In oncology, however, agency emerges as a tool-mediated achievement: preferences are not simply elicited but continually reshaped through the interaction between staging practices and communicative strategies. Autonomy, in this setting, is better understood as a tool-mediated achievement shaped by clinical tools that regulate temporal and affective orientation, rather than as a pre-given property of the patient.

#### Colorectal surgery: operative individuation and tool-mediated agency

In colorectal surgery, patient individuation can be analytically characterized as oriented toward the operative body. Here, anatomical visualization functions as a primary tool of individuation, segmenting the patient’s body into discrete, operable regions and rendering it amenable to intervention. Within this framework, the patient is configured not as an integrated psychosocial whole, but as a body organized according to the logic of surgical accessibility.

Risk quantification introduces a second axis of individuation. Probabilistic estimates do not merely convey background information; they structure the decision space by translating uncertain futures into calculable thresholds. Patients are thereby positioned as bearers of risk distributions, whose possible outcomes can be compared, weighed, and optimized within predefined parameters. Individuation in this setting is thus oriented less toward temporal trajectory than toward probabilistic configuration across alternative operative futures.

A third layer of individuation is generated through the comparative framing of surgical options. By systematically juxtaposing procedures in terms of benefits, costs, and risks, surgical reasoning scaffolds deliberation within a bounded field of possibilities. Agency, in this context, emerges not as an abstract capacity exercised independently of clinical mediation, but as an outcome of communicative infrastructures that organize how options can be understood and acted upon.

From a philosophical perspective, this surgical mode of individuation reframes autonomy itself. Rather than presupposing self-legislation by an already autonomous chooser, autonomy here is better understood as a tool-mediated achievement realized through deliberative infrastructures. Diagrams, probabilistic thresholds, and comparative framings do not simply support decision-making; they organize and stabilize the very conditions under which deliberation becomes possible. The operative body is therefore not a natural object awaiting recognition, but a constructed entity produced through the interlocking tools of anatomy, probability, and comparison. Patient-centeredness, in this model, consists in enabling agency within the decision space that these tools bring into being, rather than in appealing to a universal concept of autonomy.

#### Palliative care: experiential individuation and tool-mediated agency

In palliative care, patient individuation can be analytically characterized as oriented toward experiential subjectivity and epistemic agency. Rather than centering care on disease progression or operative success, palliative reasoning foregrounds the management of suffering, the coherence of illness experience, and the cultivation of understanding.

Symptom assessment tools, narrative practices, and informational scaffolds jointly structure this mode of individuation. Quantified symptom assessment renders suffering into modifiable variables, positioning patients as bearers of experiences that can be tracked, recalibrated, and alleviated. Narrative practices organize fragmented illness histories into coherent trajectories, thereby configuring patients as narrative subjects whose experiential continuity matters for care. Informational scaffolding, in turn, positions patients as epistemic agents whose grasp of past decisions, present conditions, and future prospects is treated as central to well-being.

Taken together, these tools generate a mode of patienthood that operates across multiple registers simultaneously: patients are individuated as suffering bodies, as narrative selves, and as epistemic agents. Patient-centeredness in this setting cannot be reduced to the recognition of a single pre-given capacity, such as autonomous choice. Instead, it consists in the ongoing work of making suffering manageable, experience intelligible, and knowledge actionable through clinical mediation.

From a philosophical perspective, this mode of individuation again complicates concept-first ideals of autonomy. Normative accounts often presuppose an already autonomous chooser capable of informed self-determination. In palliative care, however, autonomy emerges as a tool-mediated achievement: it depends on practices that dispel misconceptions, integrate fragmented experiences, and scaffold understanding over time. Autonomy here is therefore better understood as a tool-mediated achievement made possible under conditions of vulnerability.

#### From case variation to structural plurality

Placed side by side, the oncology, surgery, and palliative care models show that patient individuation is neither uniform nor incidental within our case study. Each clinic brings forth a distinctive mode of patienthood by mobilizing a particular configuration of tools-in-use. Oncology organizes patienthood as a therapeutic trajectory shaped by staging frameworks and calibrated expectation management. Surgery organizes the patient as an operative body, segmented into anatomical sites and positioned within a probabilistic decision space. Palliative care individuates patients as experiential subjects and epistemic agents, whose suffering, narratives, and understanding are central to care.

What initially appears as variability across settings is treated here as structural plurality insofar as these differences are stabilized by recurrent configurations of tools that organize reasoning in systematically different ways. By structural plurality, we do not mean any difference attributable to subspecialty goals, institutional workflow, or individual clinician style as such. A difference counts as structural, in our analytic sense, when recurrent configurations of tools stabilize what becomes salient, how reasoning proceeds, and what forms of agency become actionable across encounters within a clinical setting. By contrast, idiosyncratic communication habits, one-off institutional disruptions, or differences that do not reorganize patient individuation would not, on our usage, count as instances of structural plurality.

From a concept-first perspective, which evaluates practice by reference to a pre-given conception of the patient, such plurality is therefore likely to be interpreted as deviation from an assumed unity, even when patient-centered care is explicitly framed as responsiveness to individual patients.

Considered together, these differences suggest that what might initially appear as local variation in emphasis reflects a more systematic pattern. Across these clinical settings, different tools foreground different features, structure reasoning in distinct ways, and enable different forms of agency. From this perspective, plurality is not merely a matter of descriptive diversity, but points toward a form of structural differentiation in how patienthood is organized in practice.

This observation raises a broader metaphilosophical question. If patienthood takes different forms across clinical contexts because reasoning is structured by different tools, then the unity of patient-centered care cannot be secured simply by appeal to a single, stable concept of patienthood. How, then, should plurality be understood, and at what level should normative evaluation of patient-centered care be situated? The next section takes up this question by examining the normative implications of structural plurality for how patient-centered care is assessed and understood.

## Tool-first rethinking of patient-centered care

### From plurality to normativity

Building on the preceding analysis, this section considers what patient-centered care looks like when approached from a tool-first perspective. Rather than presupposing a single normative ideal of patienthood, a tool-first approach treats plurality as a structural feature of clinical practice: different tools individuate patients in different ways, and these differences matter for how care is enacted. From this standpoint, the normative question is not whether practice conforms to a pre-given concept of patienthood, but how tools shape visibility, agency, and responsiveness in clinical encounters.

On this basis, plurality can be understood not as deviation from an ideal but as a constitutive feature of clinical reasoning. Each practice individuates patients differently because each is mediated by distinct cognitive architectures: staging systems and expectation management, anatomical diagrams and quantified risks, symptom scales and narrative scaffolds. To understand patient-centered care, then, is not to measure practice against a pre-given definition of the patient, but to analyze how tools generate different forms of patienthood and, with them, different enactments of centeredness. On the present account, unity is better located not in conceptual essence but in clinicians’ shared responsiveness to the salience their tools make visible. It is in this sense that the tool-first reframing carries normative significance.

This shift in perspective relocates the normative task. On the present analysis, patient-centered care is not best captured as conformity to an abstract ideal such as autonomy or holism. Instead, normative evaluation is better directed toward examining how tools structure what becomes visible and actionable in practice. Questions about staging systems, risk diagrams, or symptom scales are therefore not merely technical, but ethical: they concern which futures are opened or foreclosed, which experiences are rendered salient, and which forms of agency are made possible. Normativity, in this sense, shifts from assessing conceptual fidelity to critically examining the ethical consequences of tool-mediated structures.

### Ethical ideals as tool-mediated achievements

From a tool-first perspective, ethical ideals such as autonomy, shared decision-making, and informed consent are better understood not as pre-given foundations of patient-centered care, but as achievements that emerge through clinical practice. Standard accounts in medical ethics often treat these ideals as prior normative standards that practice is tasked with implementing or respecting. By contrast, our analysis suggests that their realization depends on the cognitive, communicative, and material tools through which patients are individuated and care is organized. Seen in this light, autonomy, shared decision-making, and informed consent are sustained—or undermined—by the architectures of practice that structure how uncertainty is managed, how options are compared, and how understanding is cultivated. Ethical evaluation, on a tool-first view, is therefore not well captured as an assessment of whether practice conforms to abstract ideals. Rather, it is better directed toward examining how tools shape visibility, agency, and responsiveness in concrete clinical settings, and with what ethical consequences for patient care.

This reframing helps clarify why ethical tensions recur when patient-centered care is translated into practice. If ethical ideals are treated as fixed concepts, divergence across clinical contexts can only appear as failure, deviation, or imperfect realization. A tool-first approach instead interprets such divergence as reflecting differences in how practices are structured by distinct tools. Normativity, on this account, does not disappear; it is relocated. The critical question becomes how tools configure forms of agency, understanding, and responsibility, and whether these configurations serve or undermine the aims of care within particular clinical contexts.

This does not require abandoning ethical ideals such as autonomy, shared decision-making, or informed consent. Rather, it suggests reinterpreting them as values whose realization depends on tool-mediated conditions, and supplementing standard ethical analysis with attention to those conditions. On this view, the normative question is not only whether such ideals are affirmed in principle, but also how particular configurations of tools enable, constrain, or reshape their practical realization.

Framed in this way, the present argument opens a research agenda rather than offering a complete normative theory. A tool-first evaluation of patient-centered care would ask, at minimum, four questions: which aspects of the patient are rendered visible or obscured; what forms of agency are enabled or constrained; what trade-offs among hope, comprehension, symptom relief, and decisional responsibility are built into a given configuration; and how those configurations might be revised in light of the aims of care.

This reconceptualization also situates patient-centered care within broader debates in philosophy of science. Rather than treating normativity as external to scientific and clinical practices, a tool-first approach highlights how epistemic and ethical considerations are integrated within the instruments and procedures that structure reasoning and action. Ethical evaluation, on this view, proceeds by critically examining the design and use of tools as sites where values are operationalized in practice.^1^,^2^  

Taken together, these considerations suggest that patient-centered care is better understood not as a failed ideal or an unstructured multiplicity, but as a family of tool-mediated practices, unified not by fidelity to a single normative concept, but by responsiveness to the forms of salience and agency that tools make possible. Conclusion draws out the broader metaphilosophical implications of this view for how practices are individuated and evaluated in philosophy of science.

## Conclusion

This paper has examined how patienthood is individuated in clinical practice through tools-in-use. Drawing on an ethnographic case study of oncology, surgery, and palliative care within a single institutional setting, we have argued that patient individuation is not simply a matter of recognizing pre-given persons. Rather, it is shaped by the cognitive, communicative, and material tools through which clinical reasoning is organized. Staging systems, anatomical diagrams, risk thresholds, symptom scales, narrative practices, and informational scaffolds do not merely support practice; they help organize what counts as salient, actionable, and patient-relevant in different clinical contexts. Our claims are therefore not frequency-based generalizations about patient-centered care as such, but analytic claims about how recurrent configurations of tools can stabilize distinct modes of patienthood within a bounded clinical setting.

This analysis helps clarify why, in the present case, patient-centered care resists capture by a single normative ideal. Across the settings examined here, clinicians enact different forms of patienthood that are internally coherent yet irreducible to one another. From a tool-first perspective, the unity of patient-centered care is therefore not best located at the level of a shared conceptual essence, such as autonomy or holism. It is better understood in terms of a shared orientation toward responsiveness: clinicians attend and respond to the forms of salience that their tools bring into view, while remaining attentive to what those tools may obscure. Normative reflection, on this account, is oriented less toward conformity to abstract ideals than toward critical engagement with the structuring role of tools in practice.

The broader metaphilosophical implication concerns how practices are to be analyzed in philosophy of science. Practice-first approaches have rightly emphasized the situated and material dimensions of scientific work, but without a clear strategy of individuation they risk accumulating description without analytic focus. Concept-first approaches, by contrast, impose unity in advance, treating plurality as conceptual failure. The tool-first approach proposed here offers an alternative: it individuates practices by examining the cognitive architectures that structure reasoning in situ, and it grounds philosophical critique in reflection on how those architectures enable and constrain possibilities of action, understanding, and care. In this sense, our case study functions both as a contribution to debates in the philosophy of medicine and as an illustration of a more general strategy for analyzing practice without reducing plurality or dissolving it into relativism.

These considerations also bear on ethical reflection and policy discussions surrounding patient-centered care. Efforts to operationalize patient-centeredness through guidelines, checklists, or quality metrics often presuppose a single normative model of patienthood. The analysis developed here suggests caution toward such simplification. Where patienthood is differently organized through different tools across clinical domains, no single metric is likely to exhaust what it means to center the patient. Evaluation is better directed toward examining how specific tools shape possibilities of agency, understanding, and care within particular settings, and toward cultivating reflexivity about those effects among clinicians and institutions.

Finally, while the present analysis has focused on cancer care, the tool-first approach invites further comparative work across scientific and clinical domains. Rather than treating plurality as a problem to be resolved at the level of concepts, such work can examine how different tools stabilize different objects of practice, and how alternative tools might reconfigure what becomes visible and actionable. By attending to tools-in-use, philosophers of science can develop more precise accounts of how practices stabilize and organize their objects and how normative critique can be oriented toward the conditions that structure those practices.

## Footnotes

1 For discussions of value-ladenness in philosophy of science, see Douglas (2009) and Elliott (2017), who argue that non-epistemic values enter scientific reasoning not only at the stage of theory acceptance, but through choices of methods, evidential thresholds, and standards of risk. The present analysis extends this line of thought by examining how such values are embedded within the cognitive tools that structure clinical reasoning.

2 For philosophical accounts of pluralism, see Kellert, Longino, and Waters (2006), Chang (2022), and Massimi (2022). These authors emphasize that scientific practices often generate multiple, sometimes incompatible representations of phenomena, and that such plurality can be epistemically productive. A tool-first approach complements this literature by identifying the structural sources of plurality in practice, tracing how different cognitive architectures stabilize distinct modes of individuation.
